# New role of hypoxia in pathophysiology of multiple myeloma through miR-210

**DOI:** 10.17179/excli2018-1109

**Published:** 2018-07-04

**Authors:** Fakhredin Saba, Masoud Soleimani, Saeid Abroun

**Affiliations:** 1 Department of Hematology, Faculty of Medical Sciences, Tarbiat Modares University,Tehran, Iran

**Keywords:** multiple myeloma, miR-210, hypoxia, microvesicle, osteoblast

## Abstract

Bone is one of the most common sites of complication in multiple myeloma (MM) progression and bone remodeling gets definitively perturbed during disease progression. Hypoxia and miR-210 play an important role in hematological malignancies. In an attempt to elucidate the specificity of the pathways of hypoxia and miR-210 in suppression of osteoblastic differentiation in MM patients, we examined the effect of miR-210 and hypoxia on expression of important cytokines and genes of myeloma cells. Differentiation of BM-MSCs towards osteoblastic cells in response to microvesicles (MVs) was also investigated. Finally, we proposed a molecular model on how HIF-1α may promote bone lesions in MM patients. To validate the effect of miR-210 and HIF-1α on targeted genes, the shRNA of HIF-1α and off-hsa-miR-210 were transfected into RPMI-8226 cells. BM-MSCs were cultured in osteoblastic inducer and 50 µg/mL of MVs derived from both hypoxic and normoxic myeloma cells. We designed an *in vitro* study to establish the effects of HIF-1α and miR-210 on the crosstalk between MM and osteoblasts. We here showed that hypoxia-induced miR-210 increased the mRNA expression of VLA-4, CXCR4, IL-6 and TGF-β in myeloma cells. MiR-210 is mandatory for the hypoxia-increased resistance of MM cells to melphalan. Moreover, MVs derived from hypoxic myeloma cells substantially decreased osteoblast differentiation. Considered comprehensively, our findings explain one of the reasons of bone loss that occurs at the sites of MM and a nascent crosstalk model in MM pathogenesis.

## Introduction

Multiple myeloma (MM) is a hematological neoplasia with uncontrolled proliferation and accumulation of plasma cells in the bone marrow (BM) microenvironment (Hideshima et al., 2004[17]). Other characteristics are the presence of monoclonal antibodies in the serum and urine, kidney failure, and osteopenia (Hideshima et al., 2004[17]). Bone is one of the most common sites of complication in MM progression and bone remodeling gets definitively perturbed during disease progression (Abroun et al., 2012[1]). Devastating complications also include osteolytic lesions, bone pain, and hyper-calcemia (Abroun et al., 2012[1]). MM cells suppress differentiation and activation of osteoblast by secreting cytokines such as interleukin-6 (IL-6) and transforming growth factor-β (TGF-β) (Frassanito et al., 2001[13]; Abroun et al., 2012[1]).

TGF-β and IL-6 are known as multifunctional growth factors. They are released by MM cells and BM stromal cells (Urashima et al., 1996[38]; Gupta et al., 2001[15]; Lu et al., 2016[24]). IL-6 influences the pathogenesis of the bone disease of MM through different pathways, such as the expression of RANKL by mesenchymal stem cells (MSCs), the production of proteins involved in bone resorption, inhibition of the anti-osteoclast activity of estrogens, and reduction of bone formation (Ara and Declerck, 2010[5]). Interestingly, increased production of TGF-β correlates with increased IL-6 expression by stromal cells (Urashima et al., 1996[38]). Although TGF-β induces the proliferation of osteoblast progenitors, it potently suppresses later phases of osteoblast maturation and inhibits matrix mineralization. Hence, TGF-β plays a role in the suppression of bone formation and in the destruction of the bones in myeloma patients (Matsumoto and Abe, 2011[25]). 

The malignant plasma cells in the niche of multiple myeloma attach to stromal elements using several adhesion receptors. One of the most extensively studied adhesion molecules is CXCR4 and its receptor, stromal cell-derived factor-1 (SDF-1) (Saba et al., 2014[31]). SDF-1 is produced by osteoblast cells and its receptor, CXCR4, is expressed on myeloma cells. CXCR4 facilitates homing of MM cells to the BM and leads to adhesion of myeloma cells to the stromal cells, such as osteoblasts and extracellular matrix components; these, in turn, support the growth, survival, and progression of myeloma cells (Alsayed et al., 2007[3]; Azab et al., 2009[8]). Studies have shown that CXCR4 is involved in the cross-talk between MM cells and the BM microenvironment (Alsayed et al., 2007[3]; Azab et al., 2009[8]; Saba et al., 2014[31]). Furthermore, VLA-4 integrin plays an important role in the localization of myeloma cells to BM by interacting with its ligands, such as vascular cell adhesion protein-1 (VCAM-1) and connecting segment-1 (CS-1)/fibronectin (Uchiyama et al., 1993[36]). Consequently, up-regulation of CXCR4 and VLA-4 expression in MM might augment association of myeloma cells to bone cells.

A growing corpus of evidence has revealed that microvesicles (MVs) function as genetic messengers in intercellular communication (Belting and Christianson, 2015[9]). MVs are enclosed by a lipid bilayer and are released in the range of 200-1000 nm in diameter from the plasma membrane of different types of cells (Belting and Christianson, 2015[9]). MVs carry a variable spectrum of molecules, including DNA fragments, mRNA, miRNA, proteins, lipids, and polysaccharides to target locations or associated cells; this pattern specifically reflects the donor cell that released them (Zhang et al., 2016[43]). MVs may become sequestered in local target cells, but they can also escape over long distances for systemic distribution. Emerging evidence has demonstrated an increased release of cytokines and MVs in response to microenvironment stress, such as hypoxic conditions (Noman et al., 2016[27]). 

Hypoxia is a common feature of the BM microenvironment; regions of severe oxygen deprivation arise in the leukemia niche due to rapid cell division (Keith and Simon, 2007[23]). Hypoxia-inducible factor (HIF) family, including HIF-1 α and HIF-β, are key transcription factors of hypoxia and promote cellular adaptation of both normal and cancer cells with reduction in oxygen concentration (Keith and Simon, 2007[23]). Under hypoxic conditions, HIF-1α is accumulated in the cytoplasm and translocated to the nucleus; it heterodimerizes there with the HIF-β protein (Keith and Simon, 2007[23]). Complexes of HIF-1α/HIF1ß proteins regulate a variety of hypoxia responsive genes along with additional cofactors that bind to the hypoxia response elements (HREs) (Keith and Simon, 2007[23]). In contrast, HIF-1α is constitutively stable in myeloma cells and is expressed even under normoxic conditions (Hu et al., 2009[21]).

A large number of genes and miRNAs in myeloma cells might be the putative targets of HIF-1α (Hu et al., 2009[21]; Storti et al., 2013[34]). MiR-210 is the most consistently induced miRNA under hypoxia (Huang et al., 2010[22]). HIF1α directly binds to HREs on the proximal miR-210 promoter (Huang et al., 2010[22]). MiR-210 expression is significantly correlated to the expression of hypoxia inducible genes in myeloma cells (Annabi et al., 2003[4]). Therefore, in an attempt to elucidate the specificity of the pathways of hypoxia and miR-210 in suppression of osteoblastic differentiation in MM patients, we examined the effect of miR-210 and hypoxia on expression of important cytokines and genes of myeloma cells. Differentiation of BM-MSCs towards osteoblastic cells in response to MVs was also investigated. Finally, we proposed a molecular model on how HIF-1α may promote bone lesions in MM patients.

## Subjects and Methods

### Cell culture

Human myeloma cell lines, U266 and RPMI8226 were cultured in RPMI-1640 media (Sigma Chemical), containing 10 % FBS (GIBICO), 2 mM L-glutamine, 1 mM sodium pyruvate, 15 mM HEPES and 50 IU/ml penicillin-streptomycin humidity-controlled incubator (37 °C, 5 % CO_2_). Under hypoxic conditions, RPMI-8226 were incubated in 1 % and/or 5 % O_2_, 5 % CO_2_ using a 2 gas incubator. 

### Human bone marrow mesenchymal stem cells isolation

Bone mononuclear cells were isolated from BM aspirates by density gradient centrifugation at 435g for 30 minutes using Ficoll and seeded at 1×10^6^ cells per cm^2^ into T-75 culture flasks. The first change of medium was accomplished after 48 h isolation and adherent cells were further cultivated at 37 °C in 5 % CO_2_ atmosphere. Cells were harvested at subconfluence using Trypsin-0.02 %. Upon 70-90 % confluence, cells were detached by trypsin and thereafter replated at a mean density of 8×10^3^ cells/cm^2^ into T-75 flask in DMEM media (Sigma Chemical) containing 10 % FBS (GIBICO) (Wang et al., 2009[41]).

### Apoptosis

Apoptosis was evaluated by MTT assay and flow cytometry. Briefly, RPMI8226 (5×10^4^ cells/mL) were seeded into each well of a 96-well microplate in a final volume of 200 µL. Then 5, 10 and 20 µM of melphalan were added to RPMI-8226 cells. MTT reagent (Sigma-Aldrich) was added 24 h later and the culture was maintained for 4 h. The formazan was dissolved in DMSO and the optical density was assessed at a wavelength of 570 nm. For flow cytometry analysis, the cell apoptotic rate was detected using an Annexin V-FITC/propidium iodide (PI) apoptosis detection kit following the manufacturer's instructions (KeyGEN, Nanjing, China). Briefly, the cells were washed with PBS twice and gently re-suspended in 500 μl binding buffer containing 5 μl of Annexin V-FITC and 5 μl of PI at a concentration of 1×10^4^ cells/ml, and incubated for 15 min at room temperature in the dark.

### MiR-210 and HIF-1α knockdown

The HIF-1α loss of function was performed by transfection of lentivirus short hairpin RNA (shRNA) pool anti-HIF-1α, whereas, pLKO.1 lentiviral vector was the empty control. The HIF-1α/shRNA vector was a generous gift from Dr. Giuliani (Department of Clinical and Experimental Medicine, University of Parma; ref. Storti et al., 2013[34]). Sequences of shRNA are shown in Table 1(Tab. 1). LentimiRa-Off-has-miR-210-3p vector (anti-miR-) and pLenti-III-miR-off control vector (ABM Inc., Canada) were purchased from ABM Incorporation. Recombinant lentivirus was produced by transient transfection of HEK 293 cells following a standard protocol. Myeloma cells were infected as previously described (Storti et al., 2013[34]).

### RNA preparation and quantitative real-time PCR analysis for the expressions of bone-related genes

Total RNA was isolated from cells using TRIzol Rneasy Mini Kit purification according to manufacturer's instruction (Invitrogen). cDNA was reverse-transcribed from 5 µg of total RNA obtained from purified cells using oligo-dT primers (Invitrogen Corp). Quantitative PCR analysis was performed using the Light Cycler system (Roche Diagnostics Corp.) with SYBR green staining of DNA double strands. Cycling conditions were as follows: 10 minutes at 95 °C, 15 seconds at 95 °C, 1 minute at 60 °C for 40 cycles. The PCR primers for amplification of HIF-1α, miR-210, TGF-β, IL-6, Runx2, osteocalcin, CXCR4, VLA4 and glyceraldehyde-3 phosphate dehydrogenase (GAPDH) are shown in Table 2(Tab. 2) (References in Table 2: Molitoris et al., 2009[26]; Taghavirad et al., 2017[35]). Specificity of product was ensured by melting point analysis and agarose gel electrophoresis.

### Differentiation of BM-MSCs toward the osteoblastic cells

20 × 10^4^ human BM-MSCs were seeded in 6-well plates in DMEM supplemented with 10 % FBS, 1 % penicillin-streptomycin, 10^-8^ M dexamethasone (Sigma), 10 mM β-glycerophosphate (Sigma) and 50 μg/ml ascorbic acid (Sigma) for 21 days. To investigate the effect of MM-MVs on osteogenic differentiation, 50 µg/mL MM-MVs added to osteoblastic inducer.

### Mineralization nodule staining

To evaluate the deposition level of calcium phosphate, the mineralization nodules were stained with alizarin red S (AR-S). Briefly, the cells on slides were rinsed in PBS followed by fixation with 4 % paraformaldehyde for 10 min at room temperature. The fixed cells were incubated with 40 mmol/L AR-S (pH 4.2) at room temperature for 10 min. Cells were rinsed 5 times with PBS to reduce nonspecific AR-S staining. The cells that had red color under microscope (calcium sedimentation) were positive for osteoblast differentiation since calcium sedimentation is a marker for osteoblast maturation.

### MVs isolation 

MVs derived from RPMI-8226 cells under normoxia (pO_2_ 21 %) and hypoxia (pO_2_ 1 %) conditions were isolated by differential ultracentrifugation. Briefly, the RPMI 8226 cells (1×10^7^) were washed with PBS and incubated in RPMI-1640 media (Sigma Chemical) containing 10 % FBS (GIBICO) for 72 hours. RPMI-8226 cells were collected by centrifugation at 400×*g* for 10 min. The supernatant was then centrifuged at 7000×*g* for 20 min at 4 °C to remove the cellular debris and the resulting supernatant was centrifugation at 20000×*g *for 1 h at 4 °C. The supernatant was discarded to remove the exosomes and the MVs pellet was washed in PBS. The isolated MVs were stored in PBS at 4 °C until use.

### Dynamic Light Scattering (DLS)

Dynamic Light scattering (DLS) is one of the most commonly used techniques that frequently utilizes to estimate the size of small particles and the molecular weights of large protein complexes (Rad et al., 2016[29]). We applied DLS (Malvern Instruments) to verify the size distributions of MVs derived from MM cells. MVs were harvested from the supernatants of RPMI 8226. Then MVs size was measured using DLS method. The analyzer presented the size distribution of MVs graphically as number plots.

### Statistical analyses

The data are expressed as the mean±SEM of at least three experiments. Statistical analyses were performed using a paired Student's* t* test and one-way Anova. The differences were considered statistically significant when* P*<0.05. 

## Results

### Expression of HIF-1α and miR-210 in MM cell lines

**Figure 1:**
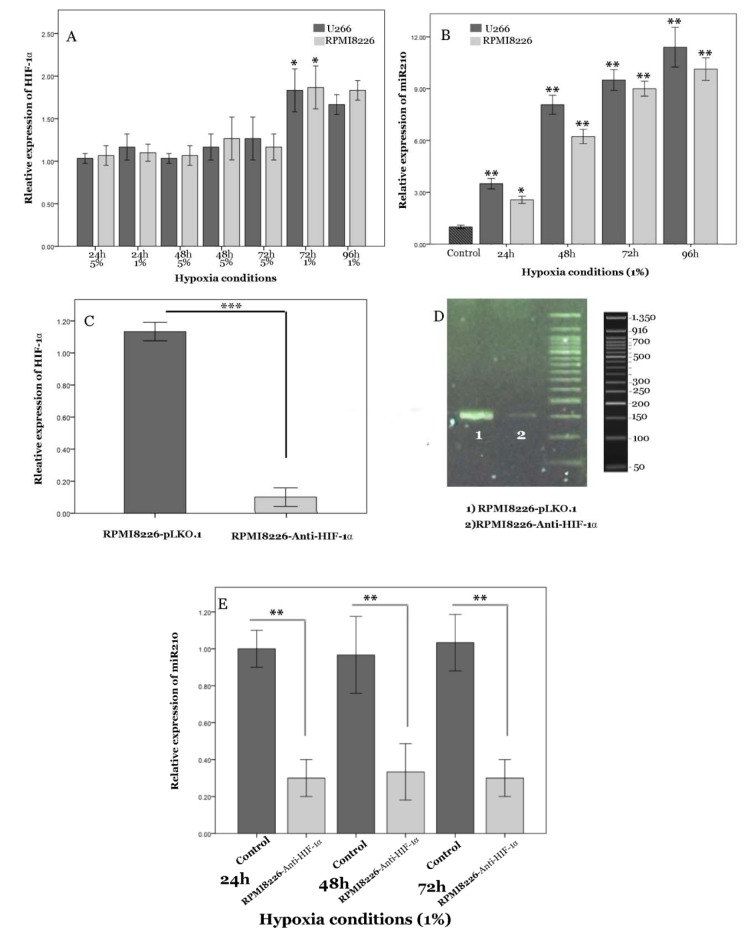
Figure1: HIF-1α and miR-210 expression by myeloma cell lines under hypoxic conditions and validation of permanent HIF-1α knockdown by shRNA. A) Relative expression of HIF-1α mRNA at different hours of hypoxia (1 % and 5 % O_2_) was evaluated in U266 and RPMI-8226. B) miR-210 expression at different hours of hypoxia (1 %O_2_) was studied in U266 and RPMI-8226. C, D) Anti-HIF-1α Lentivirus shRNA pool was used for HIF-1α stable knockdown in RPMI8226, whereas the pLKO.1 lentiviral vector was used as the empty control vector. HIF1α mRNA expression was evaluated by real-time quantitative PCR (C) and qualitative PCR (D). E) Value of miR-210 was elevated in RPMI-8226- anti-HIF-1α cells under hypoxic conditions (1 % O_2_) and RPMI-8226-pLKO.1 (control) at 24, 48 and 72 h. Internal control for all experiments was Glyceraldehyde 3-phosphate dehydrogenase (GAPDH) mRNA. Columns, mean (n=3); bars, SEM. *P<0.05, **P<0.01, ***P<0.01. Abbreviations: h (hours)

Previous studies have revealed that hypoxia specifically elevates the expression of HIF-1α (Keith and Simon, 2007[23]; Storti et al., 2013[34]). To investigate the effect of acute hypoxic conditions, including 5 % and 1 % O_2 _on HIF-1α expression, we performed real-time PCR on RNA extracted from myeloma cells. Expression of HIF-1α in RPMI8226 cells after 72 hours incubation with 5 % O_2_ showed no significant alterations (data not shown). However, HIF-1α mRNA increased under 1 % O_2_ after 72 h (Figure 1A(Fig. 1)). MiR-210, a so-called hypoxamir, is especially induced by oxygen depletion and involves different cellular pathways. We analyzed the effect of various conditions of hypoxia on the expression of miR-210 in MM cell lines, U266 and RPMI8226. The presence of 1 % O_2_ had a significant effect on basal miR-210 levels in the cell lines and caused a 10-fold increase of miR-210 expression in comparison to control (Figure 1B(Fig. 1)). However, miR-210 expression did not change significantly in 5 % O_2 _and normoxia culture conditions (data not shown). Hence, in this study, we used 1 % O_2_ as a hypoxic condition. To investigate whether HIF-1α-induced miR-210 in MM might involve the regulation of cell functions under hypoxic conditions, we studied reduced HIF-1α activity by transfection of cells with a shRNA-anti-HIF-1α (RPMI-8226-anti-HIF-1α); this was undertaken in comparison to cells transfected with pLKO.1 lentiviral vector as negative control cells. In RPMI-8226-anti-HIF-1α cells, baseline HIF-1a expression was strongly suppressed (Figure 1C, D(Fig. 1)). These results clearly indicate that shRNA-anti-HIF-1α functioned efficiently. Furthermore, a robust and stable reduction of miR-210 expression was seen even after transfection of cells with shRNA-anti-HIF-1α after 72 h of hypoxia (Figure 1E(Fig. 1)). These results suggested that the presence of HIF-1α is mandatory to elevate miR-210.

### Hypoxia modulates expression of adhesion receptors in MM 

To obtain a better understanding of the interactions between MM cells and stromal cells in the hypoxic microenvironment of BM, we evaluated the mRNA expression of CXCR4 and VLA4. Such molecules are critical for many aspects of MM biology, such as homing of MM cells to the BM environment, and survival and proliferation of malignant plasma cells. 

**Figure 2:**
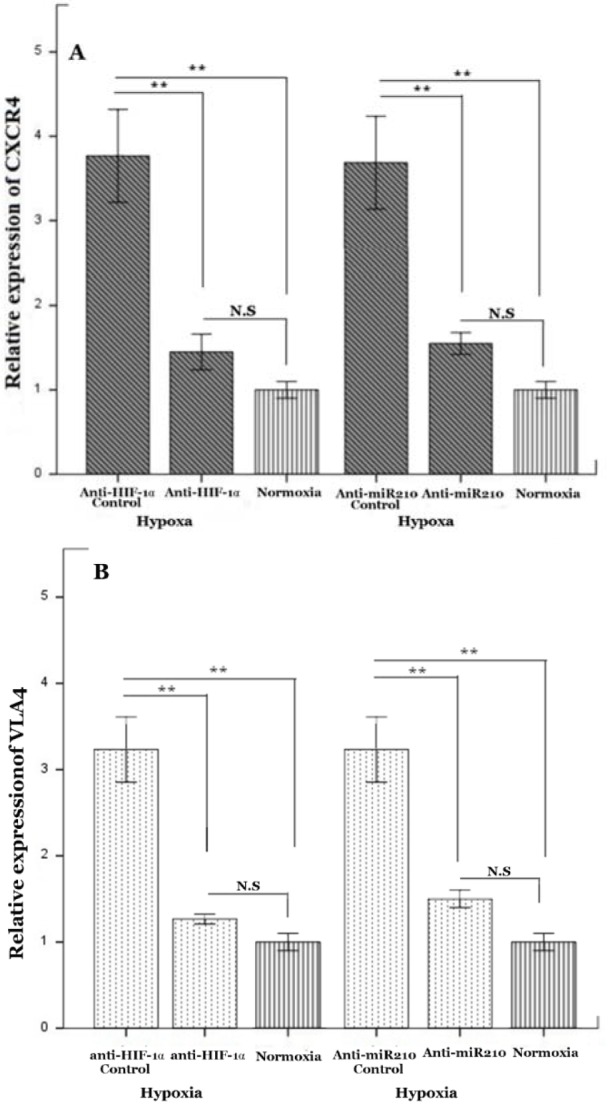
Hypoxic regulation of CXCR4 and VLA4 mRNA expression. A, B) Levels of CXCR4 and VLA4 mRNA expression were assessed in RPMI- 8226 cells using quantitative PCR after 72 h under 1 % O_2_ culture, respectively. RPMI-8226-pLKO.1 and RPMI-8226-plenti-III-off-miR210 were used as control of anti-HIF-1α, and anti-miR-210, respectively. Mean values ± standard deviation for 3 independent experiments are shown. **P<0.01. Abbreviation: N.S (not significant)

To examine the effect of hypoxia on CXCR4 and VLA4 expression in MM cells, levels of CXCR4 and VLA4 mRNA were measured in RPMI-8226 following 72 h of normoxic and hypoxic incubation. Substantial up-regulation of CXCR4 and VLA4 mRNA was observed in response to 72 h of 1 % O_2_ (Figure 2A, B(Fig. 2)). We found that cells transduced with anti-HIF-1α abrogated increased mRNA expression of CXCR4 and VLA4 under hypoxia. These findings suggest that HIF-1α is an attractive agent which increases adhesion genes in myeloma cells.

Considering that HIF-1α drastically enhances miR-210 levels in myeloma cells, we postulated that HIF-1α may affect expression of CXCR4 and VLA4 through miR-210 activation. To this end, we incubated RPMI-8226-anti-miR-210 under 1 % O_2_. As expected, no significant effect was observed on the expression of CXCR4 and VLA4 in the miR-210 knockdown cells (Figure 2A, B(Fig. 2)). Our date suggested that up-regulation of miR-210 is a prerequisite for the action of HIF-1α in myeloma cells.

### Hypoxia induces cytokines involved in bone lesion through miR-210

**Figure 3:**
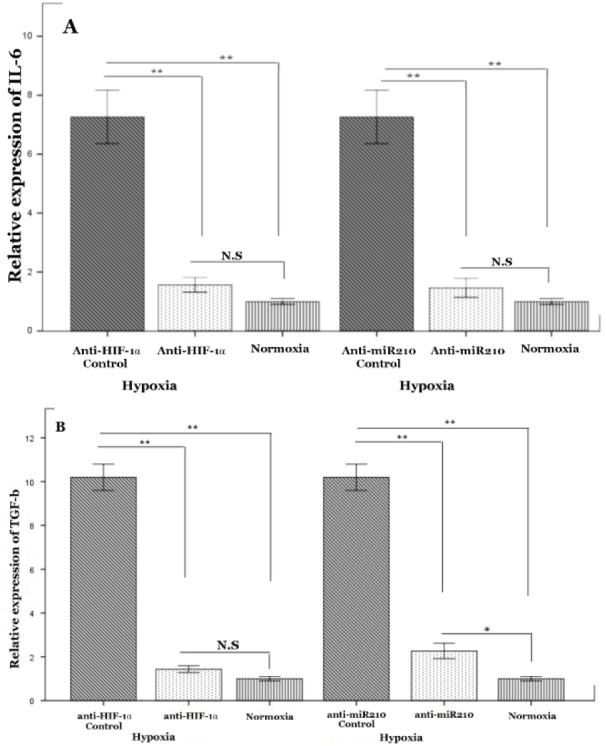
Effect of HIF-1α and miR-210 on IL-6 and TGF-β expression in myeloma cell line under hypoxic conditions. RPMI8226 cells were cultivated under hypoxia (1 % O) for 72 h. RNA was collected and subjected to quantification of IL-6 (A) and TGF-β (B) expression levels using qRT-PCR. RPMI-8226-pLKO.1 and RPMI-8226-plentIII-off-miR210 were used as control of anti-HIF-1α, and anti-miR-210 control, respectively. Mean values ± standard deviation for 3 independent experiments are shown. *P<0.05, **P<0.01, Abbreviation: N.S (not significant)

Studies revealed the potential role of IL-6 and TGF-β *in vitro *and* in vivo* growth of human myeloma cells (Edwards et al., 2008[12]). Therefore, we analyzed the influence of hypoxia on the expression of IL6 and TGF-β in MM cells. Our findings showed that, as compared to normoxia culture, MM cells were able to induce up-regulation of mRNA levels of IL-6 and TGF-β under hypoxic conditions (Figure 3A, B(Fig. 3)). To confirm the role of HIF-1α in regulating IL-6 and TGF-β of MM cells, we cultured RPMI8226 transfected with a plasmid having silenced HIF-1α and RPMI8226 with empty vector under hypoxic conditions. Inhibition of HIF-1α activity in MM cells caused a strong diminishment of TGF-β and IL-6 mRNA levels, and an almost full abrogation of hypoxia-stimulated transcription in these cells (Figure 3 A, B(Fig. 3)). We next explored the effect of miR-210 on MM cells. Similar reduction in the expression of IL-6 and TGF-β was found in the miR210-deficient myeloma cells (Figure 3 A, B(Fig. 3)). Given the fact that hypoxia promotes MM progression, it can be anticipated that a low oxygen environment fosters the accumulation of miR-210, which in turn up-regulates IL-6 and TGF-β to induce bone lesion.

### Inhibition of mir-210 enhances the sensitivity of myeloma cells to melphalan

**Figure 4:**
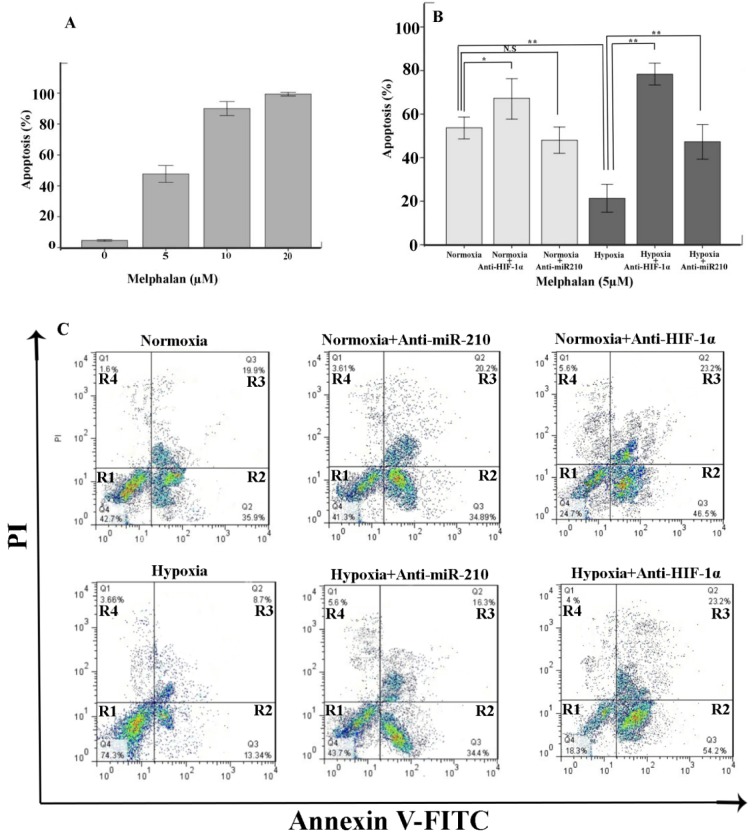
The effect of HIF-1α and miR-210 inhibition on the drug sensitivity of MM cells. A) RPMI8226 cells were cultured with the indicated concentrations of melphalan (µM) under normoxic condition for 24 h and viability evaluated by MTT test. Graph represents the mean of cells viability. B, C) The apoptosis (%) of RPMI8226, RPMI8226-anti HIF-1α and RPMI8226-anti-miR-210 was assessed, following 24 h incubation with 5 µM of melphalan under normoxic and hypoxic conditions by flow cytometry. Abbreviation: N.S (not significant). R1, viable (no‐apoptotic) cells; R2, early apoptosis; R3, late apoptosis; R4, necrosis. x‐axis: annexin V; y‐axis: propidium iodide (PI). The percentage (%) of viable cells (R1) and apoptotic cells (R2 and R3) were considered. Bars represent mean ± 95 % confidence interval of three independent experiments. *P < 0.05; **P < 0.01. Student's *t*‐test was used to test for significance.

Melphalan is widely used as a preparative drug in patients with MM. First, we treated RPMI-8226 with various concentrations of melphalan for 24 h. Then, we monitored the ratio of apoptotic cells by MTT cytotoxicity assay. Our results indicated that 5 µM of melphalan was sufficient to induce apoptosis of RPMI8226. By contrast, 10 and 20 µM of melphalan induced about 100 % apoptosis in RPMI8226 cells under normoxic conditions (Figure 4A(Fig. 4)). Based on these observations, we used 5 µM of melphalan for RPMI8226 cells throughout the study. Several lines of evidence found that hypoxia may make myeloma cells resistant to chemotherapeutic agents. However, the precise molecular mechanism of hypoxia in chemodrug resistance of myeloma cells remains unclear. Hence, we incubated RPMI-8226 cells under hypoxia for 72 h. As expected, we found that hypoxia significantly protects myeloma cells against melphalan-induced apoptosis (Figure 4B(Fig. 4)). 

We next investigated whether inhibition of HIF-1α in RPMI-8226 influences melphalan-induced cell apoptosis under both normoxic and hypoxic conditions. Previous studies have suggested that HIF-1α plays a key role in drug resistance in MM cells and that the inhibition of HIF-1α may sensitize myeloma cells to melphalan (Hu et al., 2009[21]). As expected, we found that HIF-1α suppression significantly induces sensitivity of myeloma cells to melphalan under hypoxia and normoxia conditions (Figure 4B, C(Fig. 4)). However, the effect of anti-HIF-1α on myeloma cells was more significant under hypoxia than under normoxia culture. Consequently, these findings indicate that HIF-1 is a master factor for drug resistance in myeloma cells.

Considering that hypoxia enhances miR-210 levels in myeloma cells, we treated miR-210-silencing cells with melphalan under normoxia and hypoxia cultures. We observed a significant reduction of cell viability in miR-210-deficient cells as compared to hypoxia samples (Figure 4B, C(Fig. 4)). However, the value of melphalan-induced apoptosis in RPMI-8226-anti-miR210 was of no significance under normoxia. Based on these observations, miR-210 up-regulation appears to be a prerequisite for the anti-apoptotic effect of HIF-1α in myeloma cells under hypoxic condition. 

### MVs derived from hypoxic myeloma cells suppress osteoblast differentiation from BM-MSCs

**Figure 5:**
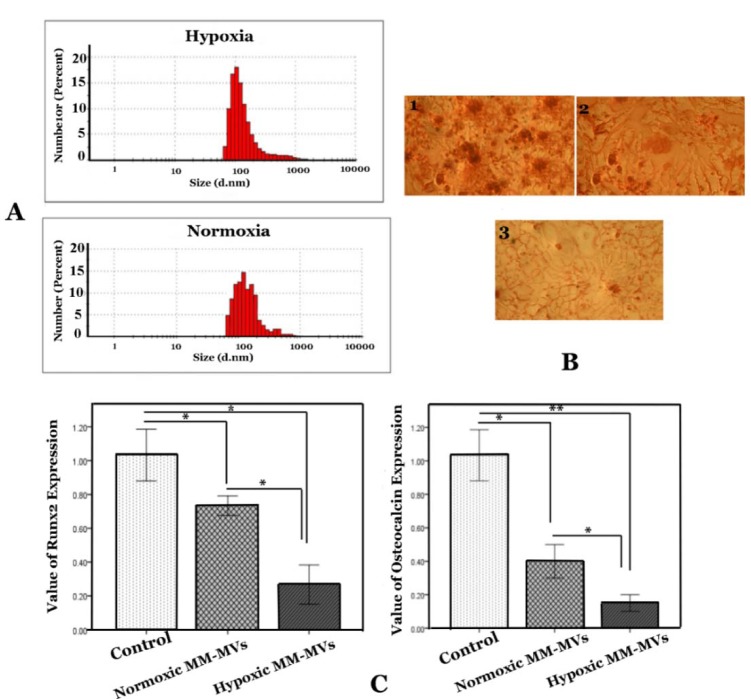
The effect of MM-MVS on osteoblasts differentiation. A) The scale bar shows size of MVs. B) Stained with alizarin red was conducted on 21 days. BM-MSCs were cultured in osteoinduction without MM-MVs (1), in the presence of 50 µg/ml MVs derived from hypoxic myeloma cells (2), and/or presence of 50 µg/ml MVs derived from normoxic myeloma cells (3). C) Results of Runx2 and osteocalcin expression in BM-MSCs that were cultured in osteogenic medium in the presence or absence of MM-MVs. RPMI-8226 cells were cultured in the normoxic and hypoxic chamber for the indicated periods (72 h). The MVs were harvested and added to osteogenic medium for 21 days. Quantification of these mRNA expressions normalized against the GAPDH mRNA levels. Data were obtained from three independent experiments and are presented as mean ± S.D. (* P <0.05, ** P <0.01).

Better understanding of the role of hypoxia will allow greater manipulation of this pathway to affect MM outcomes. MVs derived from hypoxia MM cells could be one of the paths to curtail the damage caused by osteoblasts. Therefore, MM-MVs were isolated from the conditioned medium and validated using DLS to examine size characteristics (Figure 5A(Fig. 5)). As shown in Figure 5A(Fig. 5), the scale bars indicate that these vesicles have a diameter range of 100-1000 nm. Most vesicles from the RPMI 8226 cells were 100-400 nm in size, and were, therefore, considered MVs.

To study differentiation of BM-MSCs toward osteoblast cells in the presence of MVs, we incubated BM-MSCs with MVs (50 µg/ ml) for 21 days. Our results revealed that BM-MSCs in osteogenic medium without MM-MVs were alizarin red positive after the twenty-first day; however, in the presence of MVs, BM-MSCs were not positive for alizarin red (Figure 5B(Fig. 5)). Moreover, the inhibitory effect of MVs derived from hypoxic MM cells on osteoblast differentiation was more intent than normoxic MM-derived MVs (Figure 5B(Fig. 5)).

Runx2 is an osteoblast-specific transcription factor that is necessary for the commitment of osteoblast (Adhami et al., 2015[2]). Moreover, Runx2 is an osteoblast marker which occurs after osteoblast differentiation. Therefore, we studied the effect of MVs derived from MM cells on the expression of Runx2 in BM-MSCs differentiation towards osteoblasts. Our findings revealed significantly decreased expression of Runx2 in the presence of MM-MVs (Figure 5C(Fig. 5)). However, BM-MSCs in an osteogenic medium with hypoxic MM cells derived from MVs indicated significantly higher reduction of Runx2 (Figure 5C(Fig. 5)). 

Osteocalcin is expressed by osteoblastic cells and is known as an indicator in the late stage of maturation (Zoch et al., 2016[44]). As shown in Figure 5C(Fig. 5), MVs derived from both hypoxic and normoxic myeloma cells substantially decreased mRNA expression of osteocalcin. Expression value of osteocalcin decreased severely in the presence of MVs derived from hypoxic RPMI-8226 cells (0.3 ± 0.02) as compared to MVs derived from normoxia RPMI-8226 cells (0.1 ± 0.01). Consequently, our results indicate that MVs derived from hypoxic MM are crucial regulators of BM-MSCs differentiation toward osteoblast cells.

## Discussion

In contrast to other organs, bone marrow has been accepted to be a naturally hypoxic organ. It is well known that the BM microenvironment is hypoxic in MM patients (Arendt et al., 2014[6]). During proliferation of leukemia cells, MM cells exist in an increasingly hypoxic niche which promotes disease progression (Hu et al., 2012[20]). HIF-1α is the main hypoxia factor; however, it is constitutively expressed in myeloma cells regardless of the presence of the hypoxic stimuli (Borsi et al., 2014[10]). Umezu et al. (2014[37]) demonstrated that expression of HIF-1α is overexpressed in MM cells under chronic hypoxic conditions. Similarly, we showed the up-regulated expression of HIF-1α in MM cells after 72 h of 1 % O_2_. Although a vast number of studies have examined aberrant miRNAs in MM cells, there have been few reports about effect of miR-210. This might be because miR-210 is not increased during normoxia, and because nearly all research was carried out under normoxic conditions.

Hypoxia-associated accumulation of miR-210 could switch multiple circuits of signaling in response to low oxygen conditions (Chan et al., 2012[11]). Hence, we investigated the crucial role of hypoxia and miR-210 on the pathobiology of bone degradation in MM. 

Osteoporosis is a frequent low-bone mass disorder in MM, leading to structural instability and high fracture risk (Wang et al., 2009[41]). Studies have demonstrated that differentiation and function of osteoblast are mediated by complex agents that involve signal transduction and transcriptional regulations (Wang et al., 2009[41]). In recent years, major advances in the field of myeloma have allowed better understanding of the interaction between MM cells and the BM microenvironment. Hypoxia inhibits growth, differentiation, and bone-forming capacity (Utting et al., 2006[39]). Utting et al. demonstrated that osteoblast function and bone formation are strongly dependent on oxygen (Utting et al., 2006[39]). They showed that the reduction of pO_2_ causes abolition of the bone nodule formation through decreased proliferation and differentiation of osteoblast cells to the osteogenic phenotype. Moreover, hypoxia causes a reversible state of osteoblast quiescence (Utting et al., 2006[39]). Although HIF-1α overexpression is seen in myeloma cells, the mechanism of HIF-1α in bone diseases still remains poorly understood. Accordingly, this study attempts to elucidate the effect of HIF-1α and miR-210 in the interplay of MM and osteoblast cells. 

The differentiation and activation of osteoclasts is mainly regulated by MM cells in the BM (Habibi et al., 2013[16]). Furthermore, contact between myeloma cells and osteoblasts plays a key role in regulating elevated bone lesions. Interaction of CXCR4 and SDF-1 has an important role in migration and retention of MM cells within the BM niche (Alsayed et al., 2007[3]). Low oxygen concentration induces high expression of SDF-1 and CXCR4 in different cell types (Hu et al., 2012[20]). A direct correlation between the level of hypoxia and the expression of CXCR4 in MM has been investigated (Azab et al., 2012[7]). Azab et al. showed that hypoxia increases the expression of CXCR4 in MM cells and accelerates homing of MM cells to the BM. Furthermore, SDF-1 stimulates up-regulation of VLA-4 in MM cells and modulates their trafficking and localization to the BM microenvironment (Sanz-Rodriguez et al., 2001[33]). Indeed, SDF-1 elevates both avidity and the affinity of VLA-4 to its ligands, including CS-1/fibronectin and VCAM-1 (Sanz-Rodriguez et al., 2001[33]). We showed a direct correlation between the expression of CXCR4 and VLA4 in myeloma cells with miR-210. We tested the effect of knockdown of HIF1α and miR-210 on the expression of VLA4 and CXCR4 of MM cells under hypoxic conditions. Down-regulation of HIF1α prevented the hypoxia-increased expression of VLA4 and CXCR4. MiR-210 deficient RPMI8226 showed no changes in expression of VLA4 and CXCR4 under hypoxic culture conditions. This supports the involvement of miR-210 in the regulation of CXCR4 and VLA4 during hypoxic situations. Although this needs more research, it can be hypothesized with caution that hypoxia could induce egress myeloma cells to osteoblasts through miR-210 and elevate myeloma cytokines-induced bone lesion.

TGF-β and IL-6 are considered important contributors in the MM microenvironment. IL-6 has a crucial role in the pathogenesis of MM through autocrine and paracrine mechanisms. The cellular origin of IL-6 is controversial. Several studies indicate that IL-6 is produced by the MM cells themselves (Frassanito et al., 2001[13]). Other authors, however, point to its paracrine production by BM cells and suggest that myeloma cells depend on close contact with stromal cells (Uchiyama et al., 1993[36]; Hideshima et al., 2005[18]). However, in this study we found that IL-6 expression occurs in MM cells. Studies have implicated hypoxia-stimulated IL-6 production in different types of cells (Yan et al., 1995[42]). For the first time, we demonstrated that IL-6 mRNA is significantly elevated under hypoxic culture conditions. Inhibition of HIF-1α is sufficient to overcome the hypoxia-enhanced expression of IL-6. In addition, we found that IL-6 expression is strongly down-regulated in miR210 deficient myeloma cell line; this suggests that miR210 might be essential to hypoxic-induced IL-6 expression. Interestingly, HIF-1α in MM cells is inducible by bone marrow milieu stimuli, such as IL-6 even in normoxic culture conditions (Borsi et al., 2014[10]). Therefore, a positive feedback between hypoxia and IL-6 might be considered. 

TGF-β is expressed at high levels by myeloma cells and impacts the tumor microenvironment through exacerbation of the lytic bone disease (Lu et al., 2016[24]). Previously, studies showed that hypoxia involves TGF-β response; however, there is no evidence on the effect of the mechanism of hypoxia on TGF-β in myeloma cells (Saed et al., 2000[32]). We found that miR-210 is the major regulator of TGF-β expression in myeloma cells under hypoxia. We first demonstrated that hypoxia stimulates expression of TGF-β in the myeloma cell line. Thereafter, we found decreased mRNA expression of TGF-β in RPMI-8226-anti-miR210, and TGF-β and IL-6 induced myeloma progression and bone disease (Lu et al., 2016[24]). Consequently, a knockdown of HIF-1α and miR-210 could reduce expression of myeloma cytokines-induced bone lesion.

In addition to conventional intercellular release of soluble factors, substantial attention has been given in current scholarship to the impress of extracellular vesicles being involved in the transfer of biological information in the crosstalk between cells. Wang et al. showed a novel communication mechanism between BM-MSCs and MM cells through exosome secretion (Wang et al., 2014[40]). They demonstrated that the BM-MSC-derived exosomes provide an extremely intricate compartment and support MM progression, bone disease, and drug resistance (Wang et al., 2014[40]). Moreover, MM-MVs could have mutual effects on BM stromal cells. The recent discoveries on MVs in osteoblast commitment and differentiation have opened up new approaches to understanding the pathogenesis of MM. For the first time, our data demonstrated that MVs derived from hypoxic MM cells impair osteoblast differentiation. The interplay between MM cells and osteoblasts play a crucial role in myeloma pathogenesis by extracellular vesicles. MM-MVs in the vicinity of osteoblasts might mediate bone remodeling and suppress bone formation. Recent studies have shown increased release of MVs in response to hypoxia. The exposure of myeloma cell lines to hypoxia demonstrates increase in the number of released MVs in a HIF-1α dependent manner (Noman et al., 2016[27]). Moreover, proteins and miRNAs are the core elements in MVs and the hypoxic microenvironment influences the contents of MM-MVs (Noman et al., 2016[27]; Giusti et al., 2013[14]; Penfornis et al., 2016[28]). Such a hypoxic signature carried by MM-MVs could enhance the invasiveness of tumor cells and thereby promote tumor aggressiveness. However, for the first time, we showed MVs do indeed decrease mRNA expression of Runx2 and osteocalcin. On the other hand, MVs derived from hypoxic MM acted as more substantial suppressors on Runx2 and osteocalcin expression. It has been demonstrated that MM exosomes stimulate formation and activation of osteoclasts (Raimondi et al., 2015[30]). MVs and exosomes derived from the malignant plasma cells protect osteoclast precursors, and induce osteoclasts differentiation and their bone resorption activity (Raimondi et al., 2015[30]). Increased MVs are significantly associated with decreased overall survival (OS) and could be a marker of poor prognosis in MM patients. However, the effect of MVs derived from hypoxic MM cells on prognostic factors including plasma cell percentage in the BM, β2-microglobulin concentration, and cancer stage is still not clear.

Inhibition of the HIF-1α function by a specific HIF-1α inhibitor results in enhanced sensitivity to melphalan in myeloma cells (Hu et al., 2009[21]). A combination of TH-302, a potent and highly selective hypoxia-activated prodrug, and bortezomib makes impressive improvements in multiple disease parameters and induces significantly prolonged survival (Hu et al., 2010[19]). However, we here showed that the presence of miR-210 is mandatory for the hypoxia-increased resistance of MM cells to melphalan and that the effects of melphalan increase is due to miR-210 down-regulation. Hence, targeting the miR-210 function can be used as an attractive therapeutic approach for myeloma cells.

**Figure 6:**
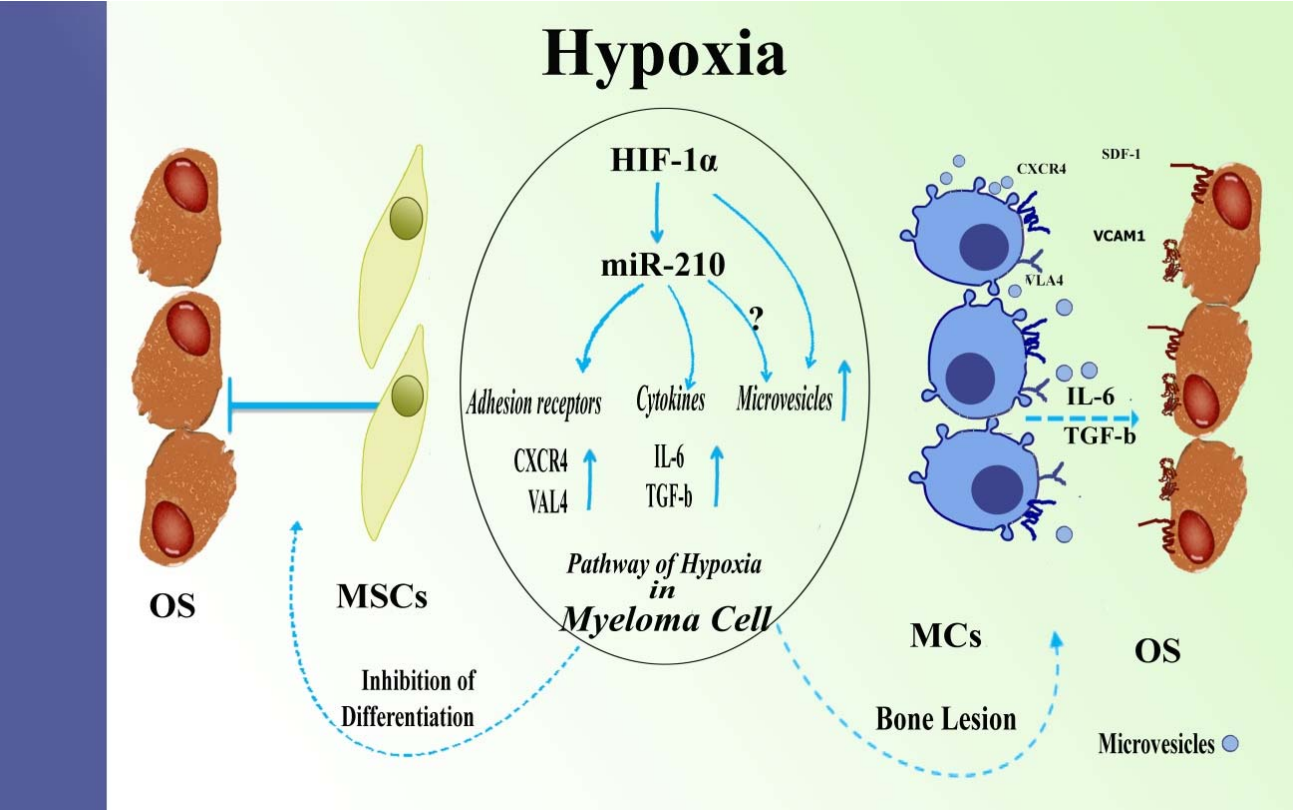
Outlook of hypoxia and miR-210 function in myeloma and osteoblast cells. Hypoxia increases expression of CXCR4 and VLA4 in MM cells, leading to augmented adhesion of MM cells to the osteoblasts. HIF-1α and miR-210 axis is elevated expression of cytokines, such as IL-6 and TGF-β in myeloma cells that progresses bone lesion. Further, MM cells could produce further microvesicles under hypoxia and MVs derived from hypoxic MM suppress differentiation of BM-MSCs toward osteoblasts. Abbreviation: OS (Osteoblast); MSCs (Mesenchymal stem cells); MCs (Multiple cells)

Considered comprehensively, our findings explain one of the reasons of bone loss that occurs at the sites of MM and a nascent crosstalk model in MM pathogenesis. We designed an *in vitro* study to establish the effects of HIF-1α and miR-210 on the crosstalk between MM and osteoblasts (Figure 6(Fig. 6)). However, the effect of miR-210 on the release mechanism of MVs is not clear and should be investigated in future studies. 

We also showed that hypoxia-induced miR-210 might augment adhesion of MM cells to osteoblasts and mediate MSCs-differentiated osteoblasts by inducing expression of IL6 and TGF-β and releasing MVs from myeloma cells. Also, hypoxia and miR-210 increase resistance of myeloma cells to chemotherapy drugs and facilitate stable cross-talk between myeloma cells and BM-stromal cells.

An important question that remains to be addressed is to understand how miR-210 could affect MVs, and how MVs derived from MM cells under hypoxic conditions may act on the signaling pathway of inhibition of osteogenesis. To address this issue, further research will be necessary to identify the signaling pathway of hypoxic MM cells on osteoblasts. Moreover, MVs might exert these effects with high specificity. Hence, specific miRNA transported to MVs functioning as genetic messengers in intercellular communication under hypoxic conditions should be investigated. Nevertheless, the role of MVs in multiple myeloma remains largely unknown

## Acknowledgement

This study was carried out with the financial support of Tarbiat Modares University, its support in providing research facilities is greatly appreciated.

## Conflict of interest

All authors declare no conflict of interest.

## Figures and Tables

**Table 1:**
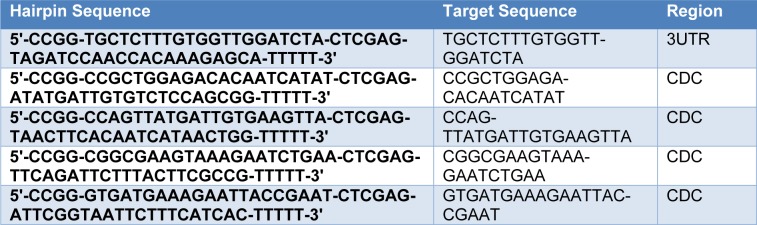
Table1: shRNA sequences of HIF-1α

**Table 2:**
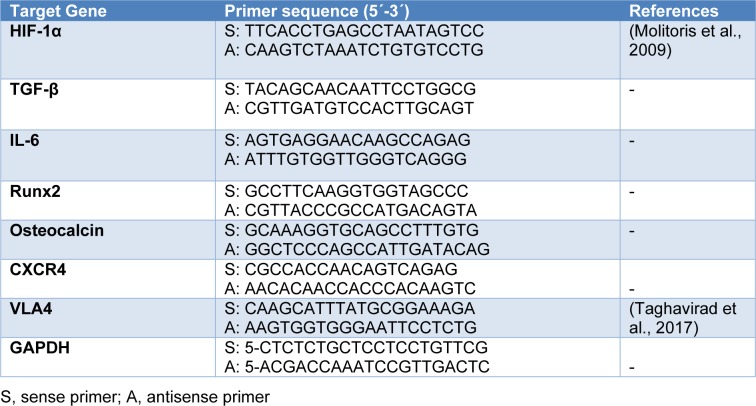
Primer sequences
